# Abnormalities in the KRAS Gene and Treatment Options for NSCLC Patients with the G12C Mutation in This Gene—A Literature Review and Single-Center Experience

**DOI:** 10.3390/biomedicines12020325

**Published:** 2024-01-31

**Authors:** Anna K. Rekowska, Piotr Rola, Agnieszka Kwiatkowska, Magdalena Wójcik-Superczyńska, Michał Gil, Paweł Krawczyk, Janusz Milanowski

**Affiliations:** Department of Pneumonology, Oncology and Allergology, Medical University of Lublin, 20-090 Lublin, Polandmagdalena.wojcik-superczynska@umlub.pl (M.W.-S.); michal.gil@umlub.pl (M.G.); janusz.milanowski@umlub.pl (J.M.)

**Keywords:** non-small cell lung cancer, *KRAS* mutations, KRAS inhibitors, immunotherapy

## Abstract

Mutations in the *KRAS* gene are among the most common mutations observed in cancer cells, but they have only recently become an achievable goal for targeted therapies. Two *KRAS* inhibitors, sotorasib and adagrasib, have recently been approved for the treatment of patients with advanced non-small cell lung cancer with the *KRAS* G12C mutation, while studies on their efficacy are still ongoing. In this work, we comprehensively analyzed *RAS* gene mutations’ molecular background, mutation testing, KRAS inhibitors’ effectiveness with an emphasis on non-small cell lung cancer, the impact of *KRAS* mutations on immunotherapy outcomes, and drug resistance problems. We also summarized ongoing trials and analyzed emerging perspectives on targeting KRAS in cancer patients.

## 1. Introduction

Kirsten rat sarcoma viral oncogene homolog (*KRAS*), a member of the rat sarcoma viral oncogene (RAS) family, is the most frequently mutated oncogene in human cancer, with a prevalence of 25–30% of all human cancer cases. The RAS protein is a small GTPase functioning as a modulator of transducing multiple growth factor receptors, such as epidermal growth factor receptor (EGFR), tyrosine kinase receptor for stem cell factor (SCF), and tyrosine kinase receptor for hepatocyte growth factor receptor (HGF) [1]. KRAS regulates the signaling pathways between activated and inactivated proteins, similarly, to an on-and-off switch. KRAS in active form initiates signaling in the mitogen-activated protein kinases (MAPK) pathway. Mutations in the *KRAS* gene lock KRAS in the active form and therefore promote downstream pathways and trigger cell proliferation [2,3].

Successful targeting of the active form of KRAS in patients with *KRAS* gene mutations became another milestone to reach; however, despite intensive research and multiple attempts, it had been considered “undruggable” until the end of 2019, when first reports of KRAS G12C inhibitors’ development hit the spotlight. Two introduced drugs, sotorasib (AMG 510) and adagrasib (MRTX849), were approved for the treatment of advanced non-small cell lung cancer (NSCLC) patients harboring the *KRAS* G12C (c.34G>T, p.Gly12Cys) mutation who have been previously treated with at least one systemic therapy. Sotorasib received FDA (Food and Drug Administration) approval in May 2021, while adagrasib received approval in December 2022 [4,5]. First observations in clinical trials revealed the effectiveness of the treatment in achieving partial response or stabilization of the disease. However, the amount of available data reporting the clinical effectiveness of KRAS G12C inhibitors is still limited. In this paper, we conducted a literature review regarding the occurrence and diagnosis of KRAS mutations and the effectiveness of various therapy methods in patients with these mutations. Moreover, we aimed to present our single-center experience in sotorasib treatment in patients with advanced G12C-mutated NSCLC from the Department of Pneumology, Oncology, and Allergology at the Medical University of Lublin.

## 2. *RAS* Gene Abnormalities in Cancers

The RAS protein superfamily includes three types of isoforms—KRAS, Harvey rat sarcoma virus (HRAS), and neuroblastoma RAS viral oncogene homolog (NRAS). RAS mutations are mostly found at codons 12, 13, and 61. The frequency, type, and location of the mutations vary within the isoforms, and those differences are linked to the development of specific cancer types [2,6,7]. Mutations in *HRAS*, responsible for 4% of all RAS mutations, are predominant in bladder and head and neck squamous cell carcinomas [8]. *NRAS* mutations account for 12% of RAS abnormalities; however, they are usually observed in cutaneous skin melanoma (24%) and acute myelogenous leukemia [2,6,9]. KRAS is the most frequently mutated RAS isoform. KRAS mutations are even present in 85% of all RAS-mutant cancers [9,10,11,12].

The KRAS gene encodes a protein with GTPase activity responsible for the control of signaling pathways leading to cell growth, maturation, and apoptosis. Mutations in the *KRAS* gene cause continuous signal transduction in the cell, leading to uncontrolled cell growth [13]. KRAS mutations occur predominantly in lung cancer (LC), pancreatic ductal adenocarcinoma, and colorectal carcinoma (CC), with an occurrence rate of 32%, 96%, and 37–52%, respectively [14,15]. Within LC patients, it is mainly detected in lung adenocarcinoma (ADC), since 13–37.2% of ADC patients carry the mutations. In comparison, it is detected in only 4.4% of squamous cell carcinoma (SCC) patients [2,16,17,18,19]. *KRAS* substitutions occur mostly in one of five hotspots in codons 12, 13, 61, 117, and 146 [9]. In NSCLC patients, G12C (glycine-to-cysteine) substitution accounts for 44–49% of all mutations in codon 12. It has been found that 12% of ADC patients, especially cigarette smokers, have the G12C mutation in the *KRAS* gene. In addition, 97% of G12C mutations in the *KRAS* gene occur in current smokers or patients with a smoking history, predominantly in women (61%). The median age of disease onset in patients with G12C substitution is estimated at 68 years [20]. However, diversity also applies to the subtype of substituted amino acid in hot spots. Therefore, G12V (glycine-to-valine) and G12D (glycine-to-aspartic acid) variants might be observed as well, with a prevalence of 18–21% and 15–18% of all substitutions in codon 12 [4,16,18,21,22].

## 3. KRAS Gene Mutation Testing and Inhibition

Distinguishing potential genomic alteration is obligatory to introduce proper treatment in NSCLC patients. According to international guidelines, next-generation sequencing (NGS) testing should at least include an examination of the following genes: *EGFR*, anaplastic lymphoma kinase (*ALK*), ROS1 proto-oncogene (*ROS1*), B-Raf proto-oncogene (*BRAF*), neurotrophic tyrosine receptor kinase (*NTRK*), RET proto-oncogene (*RET*), and MET proto-oncogene (*MET*). To date, *KRAS* testing has been a stand-alone assay, especially in advanced NSCLC lacking *EGFR, ALK*, or *ROS1* abnormalities [23,24]. However, considering KRAS inhibitors’ development and proven efficacy, current guidelines require an extension. Current recommendations suggest including the KRAS gene in a larger multigene NGS panel. It would be reasonable to also extend the panel to other frequently mutated genes such as tumor protein 53 (*TP53)*, serine-threonine kinase 11 (*STK11)*, and Kelch-like ECH-associated protein 1 (*KEAP1)* [25,26]. Unfortunately, routine use of NGS is not possible in all centers [27,28]. Real-time PCR (polymerase chain reaction) is the most used alternative, with that method characterized by greater sensitivity than NGS, while the disadvantage is the ability to identify only detailed mutations in specific areas of the genes [29]. Analysis of the mutations requires tissue specimens suitable for DNA isolation. Materials used for this purpose in non-small cell lung cancer are predominantly tissue samples from surgical resections or materials from bronchoscopy (transbronchial thin-needle aspiration biopsy, forceps biopsy) [30,31]. Interestingly, liquid biopsy (peripheral blood) is used in many aspects of LC diagnostics, including situations where other sample types cannot be collected or to determine resistance mechanisms in patients progressing to tyrosine kinase inhibitors therapy [30,32]. This method is a non-invasive alternative for collecting samples and monitoring the disease and treatment efficacy with no need for hospitalization [7,33,34]. However, molecular testing of liquid biopsy remains less sensitive than genetic testing performed on tissue material. Therefore, tissue-based testing remains the preferred method in the examination of *EGFR*, *ALK*, *ROS1*, *BRAF*, *NTRK,* and *KRAS* genes [35,36,37].

*KRAS* mutation testing in SCC is currently not widely recommended, since the *KRAS* mutation occurrence rate in this cancer subtype is low (4–8%) [18,38]. In our clinic, *KRAS* testing was performed on patients with non-squamous NSCLC, with the use of NGS (Oncomine Focus Assay) and real-time PCR. Seventy patients were tested with NGS, which enabled the detection of mutations in the *KRAS* gene in 21.4% of patients. In this group, 10% of the patients had G12C mutations: 5.7%—G12V mutation, 2.9%—Q61H mutation, 1.4%—G12S mutation, and 1.4%—G13D mutation. Real-time PCR was performed as a single assay in patients without abnormalities in *EGFR*, *ALK*, and *ROS1*. Mutations in the *KRAS* gene rarely occur simultaneously with mutations in other oncogenes [39]. Therefore, there was a higher rate of *KRAS* mutation-positive patients (33%) tested with real-time PCR. The most common mutation was G12C, which occurred in 20% of patients, while G12V occurred in 9% of patients. Other detected mutations were G12S, G12A, G13D, and Q61H. Each of these mutations occurred in 1% of patients.

The most promising outcomes have patients with the G12C mutation in the *KRAS* gene. All approved KRAS inhibitors are used in patients with this specific mutation. The first KRAS G12C inhibitor, Compound 12, was developed by the Shokat lab, but it proved ineffective in cell cultures [40]. In response to this, an improved version called ARS-853, which possesses cellular potency, was developed [40]. Regrettably, a major drawback of ARS-853 was its short metabolic plasma stability, poor bioavailability, and resistance due to mutants lacking GTPase activity, which drastically reduced rates of inhibition effectiveness [41,42]. In consideration of these limitations, a new compound, ARS-1620, was created, which proved to be potent, selective, orally bioavailable, and well tolerated in mice [43]. While these inhibitors have not found clinical applications, they have laid the groundwork for the development of more effective inhibitors included in clinical trials [14,39].

Drugs that are currently approved for use in patients with KRAS-mutant NSCLC are adagrasib (MRTX849) and sotorasib (AMG-510) [15]. However, recently published studies introduced a third G12C inhibitor, divarasib (GDC-6036), which is currently under investigation [44]. KRAS G12C inhibitors have shown promising, both in vivo and ex vivo, effects in the management of hyperactive cell proliferation resulting from *KRAS* mutation. In wild-type cells, KRAS protein alternates between a Guanosine diphosphate-bound (GDP-bound) inactive state and a Guanosine-5′-triphosphate-bound (GTP-bound) active state, whereas the mutation impairs the regulation and favors the GTP-bound form. The active state enhances a series of signaling cascades, such as phosphoinositide 3-kinase-Protein kinase—protein kinase B—mammalian target of rapamycin (PI3K-AKT-mTOR), and MAPK cascade pathways. Sotorasib and adagrasib, by selective and irreversible covalent binding to the GDP-bound state of KRAS, inhibit further downstream signaling and maintain the inactive KRAS form [3,43,45].

## 4. Effectiveness of KRAS G12C Inhibitors in Non-Small Cell Lung Cancer

CodeBreaK100 (NCT03600883) was a multi-center phase I/II trial investigating sotorasib effectiveness in locally advanced or metastatic NSCLC patients, who previously progressed on at least one systemic therapy. The 174 patients who enrolled in the study received 960 mg of sotorasib once daily. A 2-year follow-up analysis of patients treated with sotorasib showed that 41% had partial remission, the median duration of response (DoR) was 12.3 months, the median progression-free survival (PFS) was 6.3 months, and the overall survival (OS) was 12.5 months. The 2-year OS achieved 33% of patients. Long-term clinical benefit (PFS longer than 12 months) was observed in 23% of patients, regardless of the PD-L1 expression, or STK11 and/or KEAP1 co-mutations [46].

Phase III of the CodeBreaK200 trial evaluated the efficacy of sotorasib vs. docetaxel in patients with *KRAS*-mutated NSCLC who had previously been treated with platinum-based chemotherapy and checkpoint inhibitors and progressed on the treatment. The patients who had progression after docetaxel were allowed to continue treatment with sotorasib. Of patients treated with sotorasib alone, 24.8% showed 1-year PFS compared to 10.1% of patients in the docetaxel arm. The overall response rate (ORR) was 28.1% vs. 13.2% and the disease control rate (DCR) was 82.5% vs. 60.3%, respectively. There was no difference in median OS due to the cross-over of therapy. Moreover, sotorasib showed better tolerance than docetaxel [47].

The effectiveness of adagrasib was assessed in phase II of the KRYSTAL-1 clinical trial among 112 patients with *KRAS* G12C-mutated NSCLC. The ORR was 42.9% (111 patients had a partial response and 1 had a complete response). Disease control was observed in 79.5% of patients, the median DoR was 8.5 months, the median PFS was 6.5 months, and the median OS was 12.5 months [48].

Studies on both sotorasib and adagrasib excluded patients with active CNS metastases, except for patients with locally treated and stable brain metastases. However, based on sotorasib and adagrasib’s ability to penetrate the blood–brain barrier, post hoc study analyses could be performed. In the group treated with adagrasib, there were 42 patients with CNS metastases, and radiological assessment was possible in 33 of them. Median PFS was 5.4 months in all 42 patients with CNS metastases. A similar analysis was performed on the group of patients with CNS metastases treated with sotorasib. Of 16 patients who were evaluated, 2 had a complete response, 12 had a partial response, and 2 had progression [48,49].

Besides already-approved sotorasib and adagrasib therapies, multiple ongoing clinical trials have investigated other potential G12C inhibitors. Information about these studies is summarized in Table 1.

Studies investigating KRAS-positive tumors revealed that concurrent mutations can either favor better outcomes or negatively affect KRAS inhibitor therapy effectiveness. In KRAS-mutant NSCLC patients, the most frequent additional genetic alterations are mutations in the *TP53* gene (with a prevalence of 39–42%), *STK11* gene (29–30%), *Protein Tyrosine Phosphatase Receptor Type D (PTPRD)* gene (15%), and *Kelch-like ECH-associated protein 1/Nuclear Factor [Erythroid-derived 2]-like 2 (KEAP1/NFE2L2)* gene (present in 24–27% of cases) [50,51]. Narrowing the group only to patients with the G12C mutation, the most common occurring alterations are shown in the following genes: *TP53* (49%), *STK11* (23%), *CDKN2A* (10%), *ATM Serine/Threonine Kinase (ATM)* (8%), and *KEAP1* (6%) [18].

## 5. Own Experience

Lung cancer has the highest incidence among malignant neoplasms in Poland, accounting for 10% and 18% of cancer cases in women and men, respectively. At the same time, it is responsible for 6% of all deaths in Poland [52]. In 2020, 14,229 men and 8009 women died of lung cancer in Poland [53]. According to 2016 data, the lung cancer-related standardized death rate in Poland was the second highest in the European Union [54]. 

The high incidence of lung cancer cases in Poland is mainly attributed to the population of active tobacco users, since 80% of all lung cancer cases are caused by smoking [55]. In the male population, smoking prevalence started to rapidly grow from the beginning of the 20th century onwards, peaking about 30 years ago. Due to this and the decreasing number of men smoking over the last 20 years, a decreasing trend in the number of lung cancer cases among Polish men has been observed. However, the increasing lung cancer mortality rates in Polish females observed in recent years are associated with society’s demography and the aging of women born in the postwar period, when active smoking was particularly common [52].

Statistics show that more than 28% of males and 19% of females are active smokers [54]. In non-smokers, lung cancer is diagnosed much less frequently, and the incidence is estimated at less than 5 cases per 100,000 non-smoking Poles [54]. A large Polish cohort study by Lewandowska et al., apart from active and passive smoking, emphasized an undeniable correlation of occupational exposure to pesticides, asbestos, paints, and varnishes with lung cancer development [55]. Air pollution has become an emerging issue within recent decades and air quality in many Polish regions is qualified as poor. Air quality has been linked to numerous respiratory diseases, such as asthma, chronic obstructive pulmonary disease, infections, cancer, and higher mortality [56,57]. Among the 50 European Union cities with the worst air quality, 36 are in Poland [56]. 

Several particles present in polluted air, including PM2.5, PM10, and NO_2_, are considered to be carcinogenic, with an emphasis on lung cancer development [58]. Badyda et al. revealed that in 11 large Polish cities between 2006 and 2011, 20–40% of lung cancer was associated with chronic exposure to PM2.5 [59]. In 2016, 58.7% of citizens in Polish urban areas were exposed to PM10 concentrations exceeding the EU standards [58]. Moreover, Gawełko et al. revealed the role of increased NO_2_ and SO_2_ air concentrations in squamous cell carcinoma development and underlined the need for prophylactic measures for people living in regions at risk [60].

The incidence of particular types of lung cancer in the Polish population depends on the examined materials. In materials obtained during surgery, the most common type of lung cancer is adenocarcinoma, followed by squamous cell carcinoma and large cell carcinoma. However, in materials obtained from bronchoscopy, squamous cell carcinoma is most often diagnosed and adenocarcinoma is slightly less frequently diagnosed. In these materials, we observe small cell carcinoma and NSCLC not otherwise specified (NOS) relatively frequently. This is due to the location of individual types of cancers, as well as the cellularity and quality of materials collected by particular methods. Adenocarcinoma is located in the peripheral parts of the lung and is more common in postoperative findings. However, squamous cell carcinoma and small cell carcinoma are centrally located and are overrepresented in bronchoscopy materials [61].

We conducted a previous study on a group of 3856 Polish patients with NSCLC regarding the occurrence of common and rare mutations in the *EGFR* gene. In Poland, the diagnosis of *EGFR* gene mutations is performed in patients with non-squamous cell carcinoma and, until recently, only in patients with advanced NSCLC (due to the reimbursement regulations for EGFR tyrosine kinase inhibitors therapy). Approximately 8.5% of NSCLC patients described above have mutations in the *EGFR* gene. These are most often deletions in exon 19 and L858R substitution in exon 21. These mutations most often occur in young patients, non-smokers, and women. In Poland, a systematic diagnosis of mutations in the *KRAS* gene has not been carried out. KRAS inhibitors were not available in our NSCLC patients under reimbursed drug programs. The situation changed at the end of 2023. Mutations in the *KRAS* gene may be more common in the Polish population than in other Caucasian populations, due to the spread of smoking habits in patients with adenocarcinoma [62].

In our clinic, we analyzed the medical history of four patients with advanced adenocarcinoma with the G12C mutation who were treated with sotorasib between 2019 and 2022. The group consisted of one man and three women aged 61–66 years. Patients received 960 mg of Sotorasib daily. All four participants had received at least one unsuccessful systemic treatment before the sotorasib administration and fulfilled all the required remaining inclusion criteria. In our patients, *KRAS* mutations were detected by polymerase chain reaction (PCR), and the treatment was continued until disease progression or occurrence of severe adverse events (AE). A 61-year-old male patient progressed after 4 cycles of chemotherapy (first-line treatment) and 14 cycles of immunotherapy (second-line treatment). The patient has been receiving sotorasib for 16 months with disease stabilization until disease progression (PD). A 65-year-old female received 4 cycles of chemotherapy (first-line treatment) and 7 cycles of immunotherapy (second-line treatment). Sotorasib was used for 4 months until PD. The third patient was a 62-year-old female who, before sotorasib therapy, progressed on 7 cycles of chemotherapy. She achieved disease stabilization on sotorasib for 10 months. Sotorasib therapy was well tolerated in these three patients. The fourth patient, a 66-year-old female, received sotorasib as the fourth line of treatment. Due to the third-grade AE (diarrhea), the drug dosage was reduced by half, and therapy was continued for 19 months with a good response.

## 6. Acquired Resistance to KRAS Inhibitors Therapy

Various studies have attempted to find the reason behind adaptive resistance to the G12C inhibitors. Tanaka et al. discovered 10 alterations in a patient who became resistant to adagrasib therapy. Mutations observed within *KRAS*, *NRAS*, *BRAF*, and *MAP2K1* genes led to the reactivation of RAS-MAPK signaling. Behind those phenomena, Tanka et al. identified the following mechanisms: (i) activation of other RAS isoforms; (ii) other *KRAS*-activating mutations, such as G13D or G12V; (iii) potential loss of the *KRAS* G12C mutation and gain of other *KRAS* mutations; and (iv) novel mutations in *KRAS*, such as Y96D [63]. Acquired Y96D mutation directly impairs the affinity between sotorasib and residue 96, restraining G12C inhibitor compatibility and function [7,63,64]. In their study, Ryan et al. observed rapid feedback reactivation of wild-type *KRAS* following the KRAS G12C inhibition [65]. Awad et al. analyzed NSCLC, colorectal, and appendiceal cancer patients treated with adagrasib. The authors found novel acquired secondary *KRAS* mutations, including those affecting drug binding and activating cell pathways; additional activating alterations in genes encoded, such as *RTK* (receptor tyrosine kinase), *RAS*, *MAPK*, and *PI3K* (phosphoinositide 3-kinase); and acquired oncogenic gene rearrangements [34]. Interestingly, some tumors exposed to adagrasib underwent a transformation from ADC to SCC [34].

Xue et al. unveiled, in cell lines with the KRAS G12C mutation treated with novel KRAS inhibitor ARS1620, an effect of suppressed mitogen-activated protein kinase output and protein active state results from EGFR and aurora kinase (AURKA) signaling was observed. An analysis of cells exposed to ARS-1620, sotorasib, and adagrasib, that initially responded to the drugs, showed that phosphor-ERK levels returned to 75% of the initial levels within 72 h. This led to the conclusion that adaptive feedback is the main mechanism leading to KRAS inhibitor resistance [66].

Koga et al. additionally discovered that drug type and concentration determine the site of the acquired mutations. In higher adagrasib concentrations, Y69D co-mutation occurred, while O99L mutation was found in lower concentrations of adagrasib (50 vs. 100 nM). The G12D mutation was observed only in 200 nM concentration of adagrasib. Interestingly, Y96D and Y96S secondary mutations caused resistance to both adagrasib and sotorasib, and the G13D, A59S, A59T, and R68M-mutant tumors remained sensitive only to adagrasib, while Q99L-mutant cells still responded to sotorasib, despite acquired adagrasib resistance [64].

The allosteric SHP-2 (Src homology-2 domain-containing protein tyrosine phosphatase-2) inhibitors were found to be successful agents in overcoming KRAS inhibitor resistance. SHP-2 phosphatase mediates signaling between several RTKs and the active state of RAS proteins and ERK activation. SHP-2-dependent feedback loops appear to be one of KRAS inhibitor resistance’s determinants. Studies have shown that the SHP-2 inhibitor increases KRAS-GDP occupancy, which subsequently results in increased effectiveness of G12C inhibitors [15,67]. Moreover, the combination of the SHP-2 inhibitor and the G12C inhibitor modulates the immune microenvironment of the tumor via reducing myeloid suppressor cells and increasing CD8+ T cells, leading to tumor sensitivity to PD-1 (programmed death 1) receptor blockade [68]. Adagrasib and TNO155, a selective, allosteric SHP-2 inhibitor, in combination had greater activity than both agents in monotherapy [67]. In the ongoing KRYSTAL-2 clinical trial, adagrasib is combined with TNO155 in advanced NSCLC or CC patients with the *KRAS G12C* mutation. The clinical trial evaluates the safety, tolerability, drug levels, molecular effects, and clinical activity of this combination [67]. Preclinical models showed enhanced antitumor activity of sotorasib when combined with RMC-4630, another SHP-2 inhibitor. Phase 1b of clinical trial NCT05054725 had acceptable tolerability of this combination therapy in NSCLC patients, who progressed on previous therapies [69].

Combination therapy with inhibitors of SOS1 (son of sevenless homolog 1) and KRAS is another strategy for overcoming resistance to KRAS inhibitors. Inhibition of the interaction between SOS1 and KRAS protein leads to a persistent inactive GDP-bound KRAS state [70]. *STK11* and *KEAP1* gene mutations are both associated with deprived KRAS inhibitor treatment responses. If the *KEAP1* mutation was present, the response rate to sotorasib and adagrasib did not exceed 14%. If both mutations occurred supplementary to the *KRAS G12C* mutation, the rates rose to 23% and 35.7%, respectively [48,71]. Studies showed that human epidermal growth factor receptor (HER), SHP-2, mammalian target of rapamycin (mTOR), and cyclin-dependent kinase 4/cyclin-dependent kinase 6 (CDK4/CDK6) inhibitors improved adagrasib efficacy in KRAS G12C-mutant cancers [15]. This observation was confirmed in studies on patient-derived cell lines, which revealed that agents targeting RTKs and mTOR act synergistically with adagrasib, by improving tumor regression and response [72]. Amodio et al. reported reversed KRAS inhibitor resistance and an increased cell-death ratio in CC and NSCLC patients where a KRAS inhibitor and anti-EGFR antibody (cetuximab) were applied collectively [73]. Another enhancement of KRAS G12C inhibitors’ therapeutic effect was observed when treatment was combined with PI3K inhibiting agent [15].

## 7. Effectiveness of Immunotherapy in Cancer Patients with KRAS Mutations

It has been reported that in NSCLC patients with *KRAS* gene mutation, anti-programmed cell death protein 1 (anti-PD-1) or anti-programmed death ligand 1 (anti-PD-L1) immunotherapy is more effective compared to the non-mutated *KRAS* group in terms of the ORR, PFS, and OS [51,71,74,75]. Li et al. named four factors affecting immunotherapy effectiveness in KRAS-positive NSCLC patients. Among them are PD-L1 expression; existing co-mutations, such as in *TP53*, *STK11*, SWItch/Sucrose Non-Fermentable related, matrix associated, actin dependent regulator of chromatin, subfamily a, member 4 (*SMARCA4)* genes, mutation subtypes (G12C, G12V, G12D, etc.); and treatment modalities (immunotherapy, chemoimmunotherapy, other) [76]. Several studies have pointed out that the occurrence of *KRAS* gene mutations is related to an increased CD8+ T lymphocyte infiltration in the tumor microenvironment (TME) [75]. However, Mugarza et al. showed that KRAS signaling leads to the occurrence of an immunosuppressive environment by inducing cytokines and chemokines expression. Moreover, *KRAS* mutations are associated with tumor immune evasive phenotype [16].

*KRAS* mutations may also cause PD-L1 overexpression via downstream activation, increase of immunogenicity, and tumor mutation burden (TMB). PD-L1 expression and high TMB are observed more frequently if the patient has a smoking history [51,77]. Of all *KRAS* mutation subtypes, G12C is most often related to the occurrence of PD-L1 expression (65%), with high expression of this ligand (41.3%) as well as high TMB [18]. A study published by Liu et al. presented favorable therapy outcomes in patients with codon 12 mutations compared to patients with mutations in codon 13 of the *KRAS* gene [51,78].

ADC patients harboring *STK11* mutation concurring with *KRAS* mutations show lower or a lack of PD-L1 expression on tumor cells and have a lower index of infiltrating CD3+ and CD8+ T lymphocytes. Clinically, they have a weaker response to immunotherapy, with shorter PFS compared to *KRAS-*only mutant patients [25]*. STK11* mutation induces non-inflammatory TME and could be an essential factor in primary immunotherapy resistance in *KRAS-*mutated NSCLC patients, [25,79] According to Judd et al., 23% of *KRAS*-mutated NSCLC patients also have *STK11* mutations [18]. Moreover, alterations in the KEAP1/NFE2L2 pathway are associated with poorer outcomes and primary immune resistance [51]. On the other hand, patients with lung ADC with alterations in *KRAS* and *TP53* genes express PD-L1 more frequently and benefit from immunotherapy more than patients who harbor isolated *KRAS* mutation or coexistence of mutations in *KRAS* and *STK11* genes [25,51].

In NSCLC cell lines with the G12C mutation, inhibition of KRAS led to suppression of cytokines and chemokines release, particularly those leading to the recruitment and differentiation of myeloid cells. Myeloid cells in the TME may promote cancer development and progression. Therefore, they suggest that proinflammatory signals resulting from oncogenic KRAS signaling could be reversed using KRAS inhibitors. The use of KRAS G12C inhibitors caused the down-regulation of *MYC* proto-oncogene *(MYC)*, eventually leading to an increase in the interferon (IFN) signaling pathway. While *KRAS*-mutated tumors show the reduced anticancer effect of IFN, inhibition of KRAS subsequently increases the activity of type I and II interferons. Thus, secondary boosting expression of T cell chemo-attractants and antigen-presenting genes are observed [16]. Mugarza et al. observed remodeling of TME as well as an increase in T cell infiltration and activation following KRAS inhibition. Moreover, inhibition leads to up-regulation of major histocompatibility complex (MHC) class II and Cluster of Differentiation 86 (CD86) protein on dendritic cells (DCs) [16].

In tumors treated with sotorasib, Canon et al. found an increased infiltration of T cells. Moreover, the sotorasib led to an increase in the number of proliferating CD3+ T cells, and CD8+ T cells, as well as infiltration with macrophages and dendritic cells, including CD103+ cross-presenting dendritic cells [3].

Interestingly, an SHP-2 inhibitor, together with an KRAS inhibitor, modulates the TME, increases CD8+ T lymphocyte infiltration, and enhances PD-1 blockade in the in vivo models [68].

## 8. Effectiveness of KRAS Inhibitors in Other Cancers

The effectiveness of KRAS inhibitors in patients with other cancers with the G12C mutation in the *KRAS* gene compared to NSCLC patients seems less promising. In *KRAS*-mutated CC patients, the ORR for sotorasib was 9.7%, and for adagrasib, 22% [80,81]. The results of trials in the CC patients showed that more than 70% of patients achieved tumor control. The median time to progression was 5.4 months. Research into the use of KRAS inhibitors in other cancers is also in progress. So far, studies are promising, as partial responses have been obtained in pancreatic cancer, endometrial cancer, appendiceal cancer, and melanoma. The differences in the response to sotorasib therapy between NSCLC and colorectal cancer and other cancers may be a result of different predominant oncogenic factors. KRAS inhibitors’ effectiveness might be limited by the activation of EGFR, which reactivates ERK signaling. This creates the opportunity to select combination therapies of KRAS inhibitors with therapies that block relevant additional pathways [17,73].

This theory is supported by the results of a study comparing the efficacy of monotherapy with adagrasib to combination therapy with adagrasib and cetuximab. The study included 43 patients with colorectal cancer receiving monotherapy and 28 patients with this cancer receiving combination therapy. In the monotherapy group, the ORR was 19%, the median DoR was 4.3 months, and the median PFS was 5.6 months. In the combination therapy group, the ORR was 46%, the median DoR was 7.6 months, and the median PFS was 6.9 months [82].

Sotorasib has also showed promising anticancer activity and an acceptable safety profile in patients with advanced pancreatic cancer with the KRAS G12C mutation. In previously treated populations with metastatic disease, 21% of patients had partial response, the median PFS was 4.0 months, and the median OS was 6.9 months [83].

## 9. Conclusions

*KRAS* is the most frequently mutated oncogene in human cancer, contributing to approximately 25–30% of all cancer cases. *KRAS* mutations, particularly the G12C substitution, play a pivotal role in the pathogenesis of NSCLC. For decades, abnormal KRAS protein was deemed “undruggable”, causing a significant therapeutic challenge. However, in recent years, the perspective has changed with the development of KRAS G12C inhibitors such as sotorasib (AMG 510) and adagrasib (MRTX849). This article provides a comprehensive overview of the clinical characteristics of patients with *KRAS* mutations, outcomes, and ongoing clinical trials in advanced tumors with these mutations, with special consideration of NSCLC patients. Combination therapies involving KRAS inhibitors, SHP2 inhibitors, and immunotherapy are discussed as potential strategies to overcome resistance and improve treatment outcomes. This article also delves into the interplay between *KRAS* mutations and immune response. KRAS G12C inhibitors are a significant breakthrough in the treatment of advanced NSCLC and other cancers harboring *KRAS* mutations. Ongoing research and clinical trials are expected to provide further insights into optimizing KRAS inhibitors’ effectiveness and expanding their use in the evolving landscape of precision oncology.

## 10. Future Directions

Targeting KRAS G12C has succeeded and, therefore, further studies are now also investigating inhibitors in patients with *KRAS* G12D and G12V mutations [84]. Unfortunately, it was thought to be difficult to use targeted treatment for patients with these mutations, due to the chemical structure of the KRAS molecule [85]. However, drugs targeting KRAS modified by these mutations have emerged. In patients with the *KRAS* G12D mutation, MRTX1133 is being investigated. This molecule binds non-covalently to asparagine in the KRAS protein. It can bind to the active and inactive forms of KRAS. In vivo and in vitro studies have demonstrated promising effects. Currently, this drug is in the preclinical stage [86]. Phase I clinical trials, focusing on patients with the *KRAS* G12D mutation, investigate HRS-4642 (NCT05533463) and ASP3082 (NCT05382559) molecules that act by degrading KRAS protein and by inhibiting phosphorylation of ERK [87]. RMC-6236 is a RAS-selective tri-complex molecule in several phase I clinical trials, and it has been effective in targeting multiple RAS resulting from G12C, G12D, G12V, G12R, and G12X mutations, by steric inhibition of RAS binding to effectors [88,89,90]. It is worth mentioning that there are more KRAS inhibitors being investigated in ongoing preclinical studies, including TH-Z835, BI-2852, JAB-22000, and ERAS-4. All these molecules are targeting G12D [91,92].

Some drugs do not act directly by inhibiting KRAS, but by interacting with molecules that affect its activity [93]. One of these drugs is BI-1701963, an antagonist of the guanine nucleotide exchange factor for the RAS protein. This protein stimulates the release of GDP and its conversion to a GTP-binding form. Blockade of this protein can reduce the activity of downstream pathways. The drug efficacy is not related to the occurrence of a particular *KRAS* gene mutation [94]. BI-1701963 (pan-KRAS inhibitor) in combination with trametinib (MEK inhibitor) is being examined in phase I studies conducted in patients with different types of *KRAS*-mutated advanced solid tumors [95]. The phase I study enrolled patients with advanced solid tumors with a *KRAS* G12C mutation for combination treatment with adagrasib and BI-1701963 (CRYSTAL14 clinical trial) [95,96]. There are also alternative therapeutic methods for patients with *KRAS*-mutated cancers that are not based on the direct blockade of signaling pathways. Abnormal KRAS proteins are immunogenic, and this ground sparked the idea of vaccine development [97]. Peptide-based vaccines, mRNA vaccines, and dendritic cell vaccines are currently being investigated in phase I clinical trials [93,98,99,100,101,102]. Until now, there has been a lack of clinical data available for the aforementioned drugs.

## Figures and Tables

**Table 1:** Novel inhibitors in patients with solid tumors (including non-small cell lung cancer) and with the G12C mutation in the *KRAS* gene—ongoing clinical trials.

Clinical Trial Identifier	Phase	Stage of NSCLC	Setting	Treatment	Estimated Enrollment	Status
NCT04973163	Ia/Ib	Advanced or metastatic	Pretreated	**BI 1823911** **BI 701963** Midazolam	245	Active, not recruiting
NCT05132075	III	Advanced or metastatic	Pretreated	**JDQ443|** Docetaxel	360	Recruiting
NCT05119933	I/II	Locally advanced or metastatic	Pretreated or lack of treatment options	**YL-15293**	55	Recruiting
NCT05067283	I	Locally advanced or metastatic	Pretreated/Naive	**MK-1084|** Pembrolizumab| Carboplatin| Pemetrexed| **MK-1084|** Cetuximab| Oxaliplatin| Leucovorin| 5-Fluorouracil	450	Recruiting
NCT05445843	II	Locally advanced or metastatic	Naive	**JDQ443**	120	Recruiting
NCT05789082	I/II	Locally advanced or metastatic	Naive	**GDC-6036** Pembrolizumab	60	Recruiting
NCT05492045	Ib/II	Locally advanced or metastatic	Pretreated or lack of treatment options	**D-1553|**	144	Recruiting
NCT05367778	I/II	Locally advanced or metastatic	Pretreated	**HS-10370**	176	Recruiting
NCT05005234	I/II	Locally advanced or metastatic	-	**GFH925**	264	Recruiting
NCT04699188	Ib/II	Locally advanced or metastatic	Pretreated	**JDQ443** TNO155| Tislelizumab	475	Recruiting
NCT05756153	Ib/II	Locally advanced or metastatic	Naive	**GFH925** Cetuximab	45	Recruiting
NCT04956640	Ia/Ib	Locally advanced or metastatic	Pretreated/Naive	**LY3537982|** Abemaciclib| Pembrolizumab| LY3295668| Cetuximab| Pemetrexed| Cisplatin| Carboplatin	400	Recruiting
NCT05358249	Ib/II	Advanced	Pretreated	**JDQ443|** Trametinib| Ribociclib| Cetuximab	346	Recruiting
NCT05009329	I/II	Locally advanced or metastatic	Naive	**JAB-21822**	144	Recruiting
NCT05485974	I	Locally advanced or metastatic	Pretreated or not eligible for standard therapy	**HBI-2438**	44	Recruiting
NCT05002270	I/II	Locally advanced or metastatic	Pretreated	**JAB-21822|** Cetuximab	100	Recruiting
NCT04449874	Ia/Ib	Advanced or metastatic	-	**GDC-6036** Atezolizumab| Cetuximab| Bevacizumab| Erlotinib| GDC-1971| Inavolisib	498	Recruiting
NCT05999357	II	Brain metastases	Pretreated	**JDQ443**	42	Not yet recruiting
NCT05315180	I	Advanced	-	**BPI-421286**	80	Recruiting
